# Supplementary material for: Efficacy of Molnupiravir in Reducing the Risk of Severe Outcomes in Patients with SARS-CoV-2 Infection: A Real-Life Full-Matched Case–Control Study (SAVALO Study)
Source: Microorganisms. 2025 Mar 15;13(3):669. doi: 10.3390/microorganisms13030669 (PMC11944734; doi:10.3390/microorganisms13030669)
Supplement: Supplementary file 1 [file microorganisms-13-00669-s001.zip › Supplementary Table S1.pdf]

Supplementary Table S1. Number of patients stratified and outcome rates among included patients according to vaccination status.

		MNP	No MNP	p-value
Overall (n)	Vaccinated	138	1142	
	Not vaccinated	8	94	
Outcomes				
Hospital admission (%)	Vaccinated	3 (2.2)	20 (1.8)	0.730
	Not vaccinated	0 (0)	2 (2.1)	1.000
ICU admission (%)	Vaccinated	0 (0)	1 (0.1)	1.000
	Not vaccinated	0 (0)	0 (0)	1.000
Death (%)	Vaccinated	1 (0.7)	15 (1.3)	1.000
	Not vaccinated	0 (0)	8 (8.5)	1.000
Composite outcome (%)	Vaccinated	3 (2.2)	31 (2.7)	1.000
	Not vaccinated	0 (0)	8 (8.5)	1.000
