# Supplementary figures and images for: Efficacy of Molnupiravir in Reducing the Risk of Severe Outcomes in Patients with SARS-CoV-2 Infection: A Real-Life Full-Matched Case–Control Study (SAVALO Study)
Source: Microorganisms. 2025 Mar 15;13(3):669. doi: 10.3390/microorganisms13030669 (PMC11944734; doi:10.3390/microorganisms13030669)

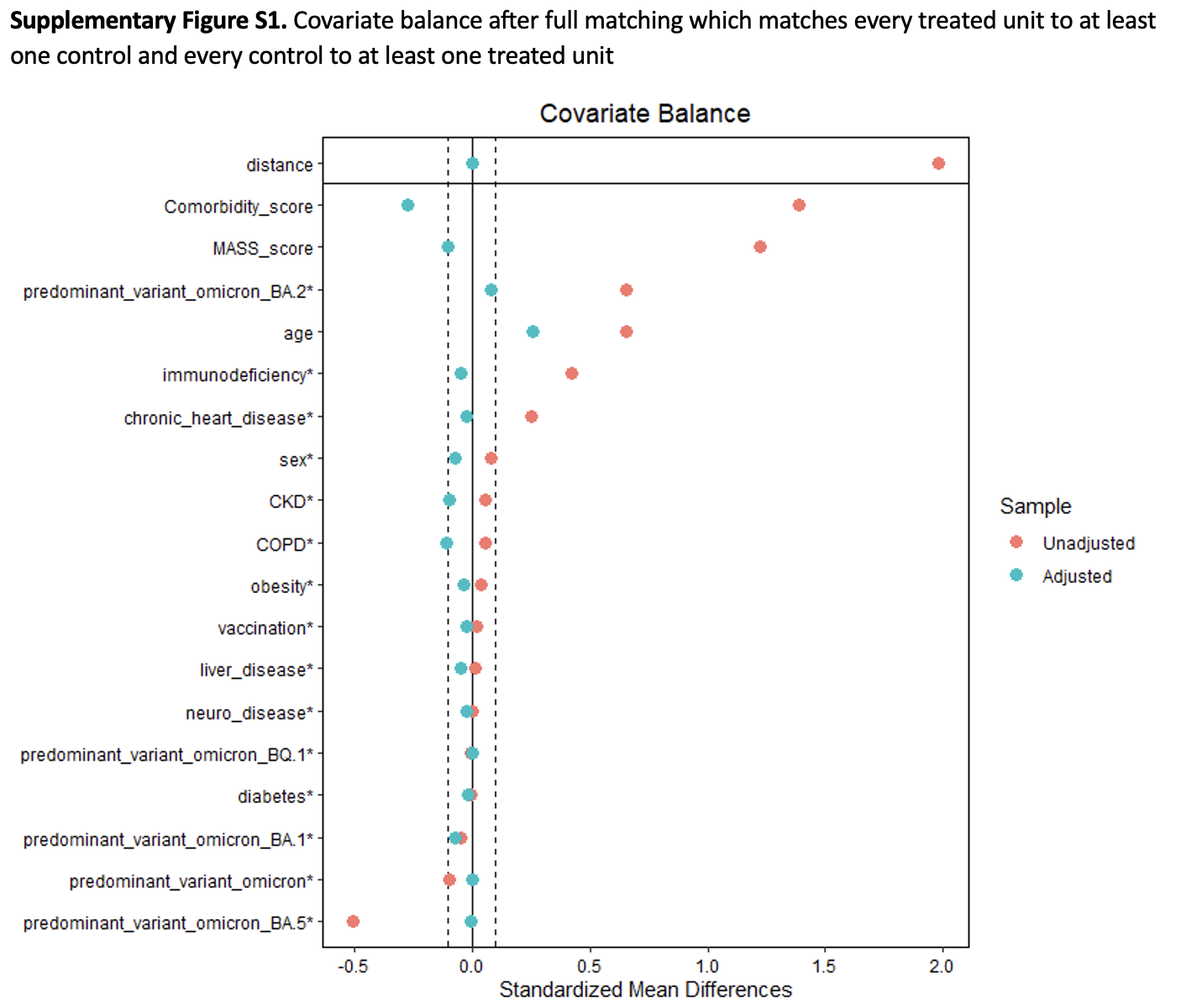

Supplement: Supplementary file 1 [file microorganisms-13-00669-s001.zip › Supplementary Figure S1.png]

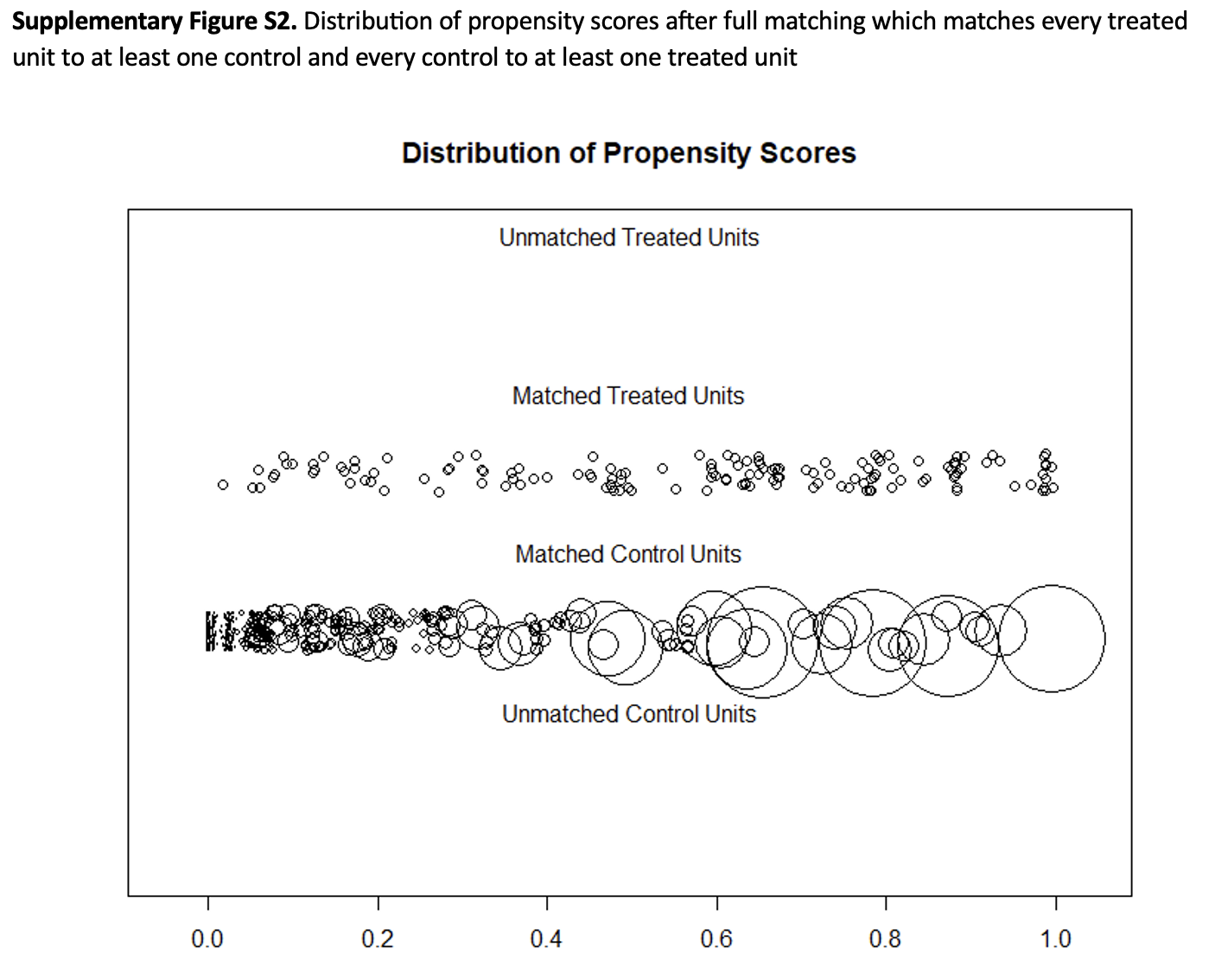

Supplement: Supplementary file 1 [file microorganisms-13-00669-s001.zip › Supplementary Figure S2.png]
